# Monitoring the Water Stress of an Indoor Living Wall System Using the “Triangle Method”

**DOI:** 10.3390/s20113261

**Published:** 2020-06-08

**Authors:** Xu Yuan, Kati Laakso, Chad Daniel Davis, J. Antonio Guzmán Q., Qinglin Meng, Arturo Sanchez-Azofeifa

**Affiliations:** 1 State Key Laboratory of Subtropical Building Science, South China University of Technology, Guangzhou 510641, China; arxuyuan@mail.scut.edu.cn (X.Y.); arqlmeng@scut.edu.cn (Q.M.); 2 Centre for Earth Observation Sciences (CEOS), Department of Earth and Atmospheric Sciences, University of Alberta, Edmonton, AB T6G 2E3, Canada; laakso@ualberta.ca (K.L.); guzmnque@ualberta.ca (J.A.G.Q.); 3 Gardens by the Bay, Singapore 018953, Singapore; chad.davis@gardensbythebay.com.sg

**Keywords:** living wall, triangle method, remote sensing, temperature, NDVI

## Abstract

Living walls are important vertical greening systems with modular prevegetated structures. Studies have suggested that living walls have many social benefits as an ecological engineering technique with notable potential for reconciliation ecology. Despite these benefits, there are currently no mature workflows or technologies for monitoring the health status and water stress of living wall systems. To partially fill the current knowledge gap related to water stress, we acquired thermal, multispectral, and hyperspectral remote sensing data from an indoor living wall in the Cloud Forest of the Gardens by the Bay, Singapore. The surface temperature (Ts) and a normalized difference vegetation index (NDVI) were obtained from these data to construct a Ts-NDVI space for applying the “triangle method”. A simple and effective algorithm was proposed to determine the dry and wet edges, the key components of the said method. The pixels associated with the dry and wet edges were then selected and highlighted to directly display the areas under water-stress conditions. Our results suggest that the proposed algorithm can provide a reasonable overview of the water-stress information of the living wall; therefore, our method can be simple and effective to monitor the health status of a living wall. Furthermore, our work confirms that the triangle method can be transferred from the outdoors to an indoor environment.

## 1. Introduction

Vertical greening systems, the result of plants being attached to buildings using different kinds of supporting mechanisms, is one way to improve the environmental conditions of urban areas [1]. The systems can be classified into green facades and living walls [2,3] based on their architecture [4]. Green facades are built using climbers supported by various mechanisms (e.g., steel cables or trellises; [4]). Living walls, also known as green walls and vertical gardens, are modular prevegetated structures where each module contains a growing medium (e.g., soil; [1]) and piping systems for irrigation [2]. These systems can be continuous or modular based on their supporting structure. Continuous systems have lightweight screens where plants are potted individually, whereas modular systems have a growing substrate in a fixed vertical structure [5].

The living wall system is an ecological engineering technique with notable potential for reconciliation ecology, a concept of modifying the anthropogenic environment in ways that encourage nonhuman use [6]. These systems can also have many social benefits, such as reducing greenhouse gases, thus helping to mitigate the effects of climate change, as well as the ability to improve air quality and indoor and outdoor comfort conditions [2,7,8,9]. Sheweka and Magdy discussed the significance of living wall systems in addressing climate change and energy crisis, as well as their function for urban agriculture, urban gardening, and art [10]. Perini and Rosasco used the cost-benefit analysis to demonstrate that some living wall systems are economically sustainable in the long run [1]. Mazzali et al. monitored some living wall systems to determine their thermophysical behavior and to analyze their effects on reducing the building energy [11]. The results conform to those by Ottelé et al. [8], Perini et al. [12], Chen et al. [13], and Razzaghmanesh [14], suggesting that living wall systems can provide notable energy savings in the indoor space as a consequence of reducing the need for heating and cooling. Due to the effect of attenuating sound energy, living wall systems can also provide acoustic benefits for the buildings [15]. 

Living wall systems require regular irrigation and fertilization to be healthy and sustainable [16]. Due to the vertical characteristics of living wall systems, artificial irrigation is considered to be mandatory to avoid water stress of the attached plants [17]. Water stress can result in various responses in biochemistry and physiology of the plants (e.g., water-loss in plant cells; [18] and limitation in photosynthetic capacity; [19]). Severe water stress is an important factor that can affect plant health [20]. Despite the benefits of living wall systems, there are currently no established workflows to monitor their health status. Sensors have been suggested for the regular monitoring of living wall systems [5], but due to the need of maintaining and protecting such systems from theft and vandalism, they may not be optimal for all living wall environments. 

Here, we study the applicability of the “triangle method” to obtain information on the health status of plants attached to a living wall system; specifically, water-stress. The triangle method, first introduced by Price [21], has been named after the triangular or trapezoidal shape of the scatter plot of the surface temperature (Ts) against a vegetation index (VI). The VI most commonly used is the normalized difference vegetation index (NDVI: First applied and reported by Rouse et al. [22]), albeit enhanced vegetation index (EVI; [23]) and fractional vegetation cover (Fr; [24]) have also been used. In the triangle method, the changes in the Ts for a given VI are assumed to be caused by the evaporative cooling effect [25]. Due to this effect, the shape of the Ts-VI space results from the intrinsic properties of vegetation: The range of surface radiant temperatures decreases as the vegetation cover increases, as a result of which the Ts-VI space typically has a triangular shape [26]. In general, vegetation temperatures are expected to have a narrower range than soil temperatures due to latent heat transfer [27], resulting in the narrow apex of the Ts-VI triangle space. In this space, there are two limiting edges: The warm/dry edge and the cold/wet edge [28], herein called the “dry edge” and “wet edge” for simplicity. The dry edge defines the maximum water stress where the surface soil water content is low and evapotranspiration is minimal [29]. On the contrary, the wet edge represents areas of readily available soil moisture and potential evapotranspiration [29]. An accurate determination of dry and wet edges is crucial to a successful implementation of the triangle method [24].

The high temperature values of water-stressed vegetation stem from the closure of plant stomata, which results in a need to dissipate more energy as sensible heat [30]. However, even in the environments of water stress, zero evapotranspiration rarely occurs due to the root zone soil water uptake of plants [31]. Unlike the surface temperature, the VI is insensitive to soil moisture changes due to a time lag before water stress becomes visible in leaves [23]. Temperature differences of the Ts-VI triangle mainly reflect temperature changes in the soil short of wilting vegetation [26]. An important assumption of the triangle method is that the triangular or trapezoidal space is caused primarily by the water availability rather than atmospheric conditions [31]. Therefore, the application of the triangle method requires uniform atmospheric conditions.

In previous studies, the triangle method has been applied to remote sensing data (commonly satellite data, e.g., the moderate-resolution imaging spectroradiometer MODIS Terra) to estimate evapotranspiration (e.g., [32,33,34,35]) or soil moisture (e.g., [36,37,38]). Evapotranspiration and soil moisture are essential parameters to understand hydrologic cycles, climate dynamics, and terrestrial ecosystem productivity [39,40,41], and thereby are significant in various applications such as agriculture [42]. In addition, the triangle method can also be applied to other fields, e.g., the estimation of fractional vegetation cover and the instantaneous surface energy fluxes [43], the assessment of future drought risk [44], and the analysis of the temporal and spatial changes influenced by the groundwater depression cone [45].

In this paper, we explore the applicability of the triangle method to monitor the water-stress of an indoor living wall system. To this end, we acquired thermal infrared, multispectral, and hyperspectral imagery from one of the largest living wall systems in the world, the Cloud Forest of the Gardens by the Bay, Singapore. The triangle method was then applied to quickly obtain and display the water-stress information of the Cloud Forest plants as a proxy of their health status. 

## 2. Materials and Methods

### 2.1. Study Area and Data 

**Figure 1:**
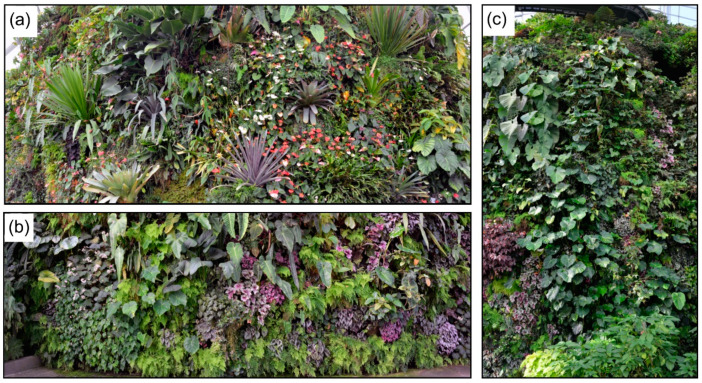
Photos of the different sections of the living wall. (**a**) Site A; (**b**) Site B; (**c**) Site C. Different types of plants from tropical mountain areas are distributed on the living wall.

This study was conducted in the Cloud Forest of Bay South Garden at the Gardens by the Bay (1°17′ N, 103°52′ E), located in the southern region of Singapore. The 1.01 km^2^ Gardens by the Bay is planted on land reclaimed from the sea [46], comprising three distinctive waterfront gardens: Bay South, Bay East, and Bay Central [47]. Of these gardens, Bay South is the largest with 0.54 km^2^. Bay South has two large conservatories, the Flower Dome and the Cloud Forest. The Cloud Forest Conservatory is a 0.8 hectares space including one of the largest and most diverse living walls in the world. The 42 m tall living wall covers 0.38 hectares of vertical planting space with more than 60,000 plants of approximate 500 taxa (e.g., ferns, bromeliads, and begonias; Figure 1). 

**Figure 2:**
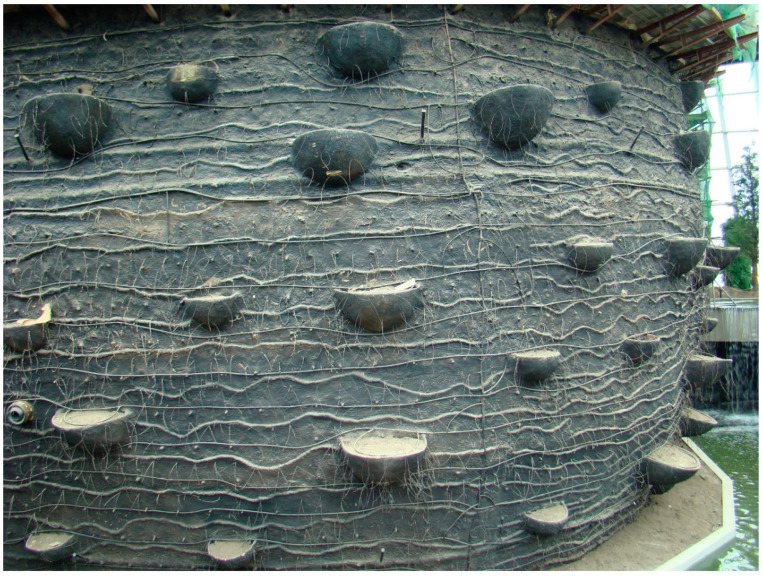
The basic structure of the living wall. The structure is a block building with a mixture of concrete, clay, and organic material (peat) applied as 2 inches of thick slurry over a wire mesh attached to the exterior. The pop-out baskets are designed to provide space for the soil and root.

The basic structure of the living wall is a concrete block building with a textured rendering (Figure 2), a mixture of concrete, clay, and organic material (peat), attached to the exterior. Hundreds of pop-out baskets are on the block building to provide soil and root space. The plants attached to the living wall are predominantly epiphytes and lithophytes as their roots can grip on and in some cases into the rendering, and thus are well adapted to live on the living wall.

The irrigation of the living wall is supplied via three different automated systems based on schedules, as described in the following. First, drip-lines with regulated emitters (set at every 30 cm) were placed in horizontal bands across the surface of the living wall. The distances between two adjacent pipes are 30 cm. Second, pop-up rotary sprinklers were placed in specific areas to meet the needs of certain plants (e.g., large bromeliads) that need to collect and hold water on their leaves. Third, high-pressure misting lines were placed strategically to maintain high humidity levels and provide some supplemental water. The fertilizer is supplied via the same network of drip and sprinklers.

The climate of the conservatory is emulated as the cool-wet conditions of tropical highland regions between 1000 and 3000 m above sea level [48]. The indoor air temperature of the conservatory ranges from 16 to 28 °C and the humidity ranges from 50% to 90%. The conservatory has over 0.45 hectares of vegetated areas and is fully enclosed by a transparent glass curtain wall. The glass facade is a double pane with a low emissivity (“Low-E”) coating that allows in the photosynthetically active light, while reducing the amount of ultraviolet and blocking much of the infrared light that would carry heat into the domes. 

To test the applicability of the triangle method to a living wall system, three kinds of data were acquired on 5 November 2018 in the Cloud Forest conservatory: (i) Thermal infrared, (ii) multispectral, and (iii) hyperspectral imagery. Considering possible differences on micrometeorology (specifically relative humidity and ambient temperature) in the dome, the data were acquired in three locations inside the conservatory: Lower level (Site “A1” and “A2” from herein), middle level (Site “B1” and “B2” from herein), and upper level (Site “C1” and “C2” from herein). In each site, the three kinds of data were acquired from the same sections of the living wall. The area covered by the remote sensing imagery was a subsection of the larger living wall areas shown in Figure 1 for each site. A high-resolution visualization of the selected living wall can be found at http://gigapan.com/gigapans/212293. 

The thermal infrared data were acquired using the FLIR T400^®^ (FLIR Systems, Wilsonville, OR, USA) camera that has one band between 7.5 and 13 µm. This camera collects data with an image resolution of 320 × 240 pixels and a field of view (FOV) of 25 × 19°. The FLIR T400^®^ camera has a thermal sensitivity of 0.05 at 30 °C and an error of 2% (e.g., 2% of 30 °C = ±0.6 °C). 

The multispectral data were acquired using the Tetracam ADC^®^ (Tetracam Inc., Chatsworth, CA, USA) camera. This camera collects data with an image resolution of 2048 × 1536 pixels and a FOV of 39.3 × 31.6°. The Tetracam ADC^®^ camera has a single precision 3.2 megapixel CMOS sensor and three bands: The green (0.52–0.60 μm), red (0.63–0.69 μm), and near-infrared (0.76–0.90 μm).

The hyperspectral data were acquired using the Specim IQ^®^ (Specim–Spectral Imaging, Oulu, Finland) camera. This camera has a spectral resolution of 7 nm and 204 bands across the wavelength range of 397–1003 nm. The Specim IQ^®^ camera acquires data with a spatial sampling of 512 pixels such that each image is a square of 512 × 512 pixels. The fore optics is designed to provide a 31° FOV. The hyperspectral data were acquired only in Site C1 and C2 for reference purposes. The integration time, one parameter set in the Specim IQ^®^ camera in the hyperspectral data acquisition, was 1 millisecond for both Site C1 and C2.

These cameras were mounted on a tripod at a 1.5 m height and a variable distance (4.3–6.5 m measured using a laser ranger, see Table 1) from the target areas of the living wall. The distance was not constant due to the variable site geometry and obstacles (e.g., groups of people) in the conservatory. The lower level of the living wall has the lowest air temperature (Site A1-2: 18.1–18.7 °C), whereas the middle level has the highest (Site B1-2: 21.6 °C). The air temperature of the upper level is moderate (Site C1-2: 20.5–20.6 °C). The data acquisition for all the sites was accomplished in around 1 h (between 09:25:00 AM and 10:30:00 AM).

### 2.2. Methods

#### 2.2.1. Data Preprocessing

A teflon panel with reflectance of 99%, positioned in the FOV of the cameras, was used to normalize the multispectral and hyperspectral data. The normalization (conversion from raw data to reflectance data) was conducted using the PixelWrench2 (version 1.1.6757.22925, Tetracam Inc.) software and the in-build software of the Specim IQ^®^ camera for the multispectral and hyperspectral data, respectively. The temperature values acquired by the thermal infrared camera were adjusted by the atmospheric temperature, humidity, and sensor-target distances (for details, see Table 1) using the FLIR Tools software (version 5.13.18031.2002; FLIR Systems), as a result of atmospheric attenuation and emittance, and an emissivity value of 0.97 was used as the standard to correct all thermal imagery. 

Next, thermal infrared, multispectral, and hyperspectral data were coaligned to enable direct comparisons between these datasets. In practice, this was done by warping the thermal infrared and hyperspectral data using the feature points of the master data (multispectral data). Here, multispectral data were chosen to be the master data due to its highest spatial resolution. Thus, it was ensured that the spatial resolution of the datasets will not be coarsened in the coalignment process. Due to the varying FOVs of the thermal infrared, multispectral, and hyperspectral instruments, the images cover slightly different areas even when taken from the same spot. To conduct the comparisons and data analysis, the intersections of the three images were extracted from them after the coalignment process. These extracted images (six thermal infrared images, six multispectral images, and two hyperspectral images) of the six sites were further used in the data analysis. For the purpose of distinction, the results associated with the hyperspectral data in Site C1 and C2 were named C1HSI and C2HSI, respectively.

#### 2.2.2. Data Analysis

First, NDVI values were calculated using all of the multispectral and hyperspectral images. For multispectral images, this was done using the built-in “NDVI” functionality of the PixelWrench2 software. For hyperspectral images, this was done using the “Broadband NDVI” functionality of the ENVI software (L3Harris Geospatial, Melbourne, FL, USA; [49]). To separate pixels associated with vegetation from pixels not associated with vegetation, we left out pixels with NDVI values of <0.2 of the multispectral and hyperspectral images for further analysis, following Sobrino and Raissouni, Sobrino et al., and Jiménez-Muñoz et al. [50,51,52] who classified pixels with an NDVI of <0.2 as soil or sparse vegetation. Based on a visual analysis of the images, such pixels represent the construction materials of the living wall system. 

To compare with the results of the multispectral data, endmember extraction and linear spectral unmixing were applied to the hyperspectral data. This process was conducted to ensure that data analysis was conducted only on the purest pixels associated with vegetation, containing mainly two steps. First, the bands in the range of 757–778 nm and above 802 nm were removed due to oxygen absorption [53] and low signal-to-noise ratios, respectively. Therefore, a total of 75 bands were removed and further analysis was conducted using the remaining 129 bands. Second, the Sequential Maximum Angle Convex Cone (SMACC) endmember extraction algorithm, based on a convex cone model for representing vector data [54], was applied to the hyperspectral images to find the purest representatives of vegetation. At this step, 30 endmembers were generated for each site, from which 8 and 5 endmembers were chosen for Site C1 and C2, respectively. These 13 endmembers, selected based on expert knowledge on the typical spectral shapes and spatial patterns of vegetation, were given as input to the SMACC algorithm to extract the purest pixels associated with vegetation. These pixels were then used in the subsequent data analysis.

Next, a correlation analysis was conducted to study the relationship between temperature and NDVI values. Here, the Kolmogorov-Smirnov test [55] was first conducted to explore the distribution of the two variables. According to the results of the Kolmogorov-Smirnov test (*p* = 0), the temperature and NDVI values are nonnormal distributed for all the sites. Thus, the Spearman’s rank correlation coefficient was chosen and calculated to conduct the correlation analysis based on its applicability [56].

Afterwards, scatter plots of temperature and NDVI were created to construct the Ts-VI space of the triangle method. In this process, a kernel smoothing function known as density estimation [57] was applied to investigate the density distribution of the scatter points. Here, the Ts-VI spaces were constructed by setting the temperature as an abscissa and VI as an ordinate, following Carlson [26]. Next, inspired by the study conducted by Sun et al. [58] and Long and Singh [28], we determined the dry and wet edges according to the spatial distribution of the scatter points in three steps, which is described as follows:

(1) All scatter points are sorted first based on the NDVI value and then based on the temperature value in an ascending order;
(2) For the given maximum NDVI value ($NDVI_{max}$), the scatter point with the maximum temperature value ($Ts_{max,NDVI_{max}}$) is determined as the top endpoint of the dry edge; the scatter point with the minimum temperature value ($Ts_{min,NDVI_{max}}$) is determined as the top endpoint of the wet edge. For the given minimum NDVI value ($NDVI_{min}$), the scatter point with the maximum temperature value ($Ts_{max,NDVI_{min}}$) is determined as the bottom endpoint of the dry edge; the scatter point with the minimum temperature value ($Ts_{min,NDVI_{min}}$) is determined as the bottom endpoint of the wet edge.
(3) After the four endpoints have been determined, i.e., Point Dry_top_ ($Ts_{max,NDVI_{max}},\;NDVI_{max}$), Point Dry_btm_ ($Ts_{max,NDVI_{min}},\;NDVI_{min}$), Point Wet_top_ ($Ts_{min,NDVI_{max}},\;NDVI_{max}$), and Point Wet_btm_ ($Ts_{min,NDVI_{min}},\;NDVI_{min}$), the dry and wet edges are calculated according to the coordinate values and displayed as Equations (1) and (2), respectively.

(1) $$f{\left(x\right)}_{dry}=\frac{x-Ts_{max,NDVI_{max}}}{Ts_{max,NDVI_{min}}-Ts_{max,NDVI_{max}}}\left(NDVI_{min}-NDVI_{max}\right)+NDVI_{max}$$

(2) $$f{\left(x\right)}_{wet}=\frac{x-Ts_{min,NDVI_{max}}}{Ts_{min,NDVI_{min}}-Ts_{min,NDVI_{max}}}\left(NDVI_{min}-NDVI_{max}\right)+NDVI_{max}$$

Finally, the scatter points beyond the dry and wet edges were selected as the points of the two boundaries, and marked using red and blue colors, respectively. According to the interpretation of the dry and wet edges by Tang et al. [24], the red points represent the areas with water-stress conditions, whereas the blue points represent the areas with sufficient water conditions. To visually display these marked areas, the scatter points were corresponded to the pixels of the multispectral images. Thus, these images can provide the water-stress information of the plants directly and be used to monitor the health status of the living wall.

## 3. Results

### 3.1. Data Distribution and Correlation Analysis

**Figure 3:**
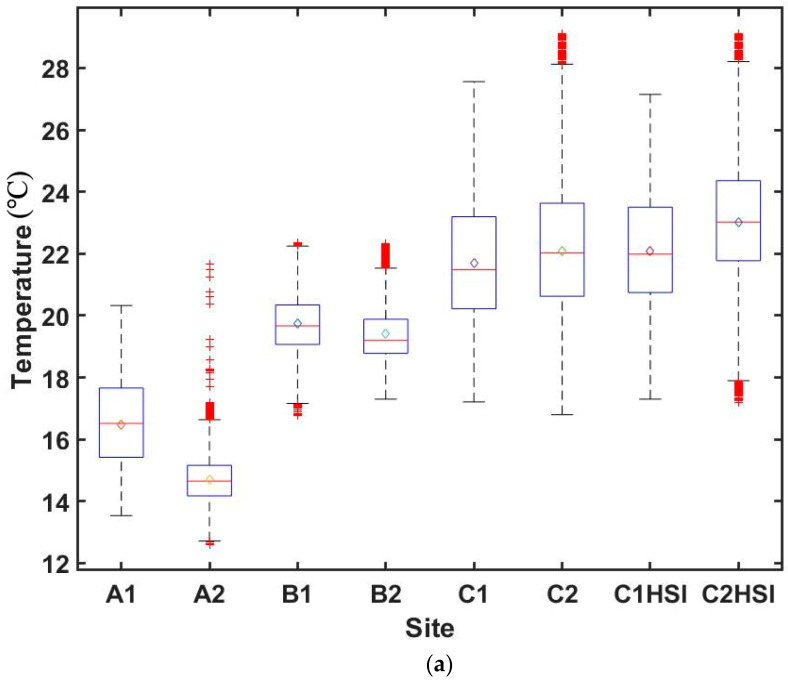

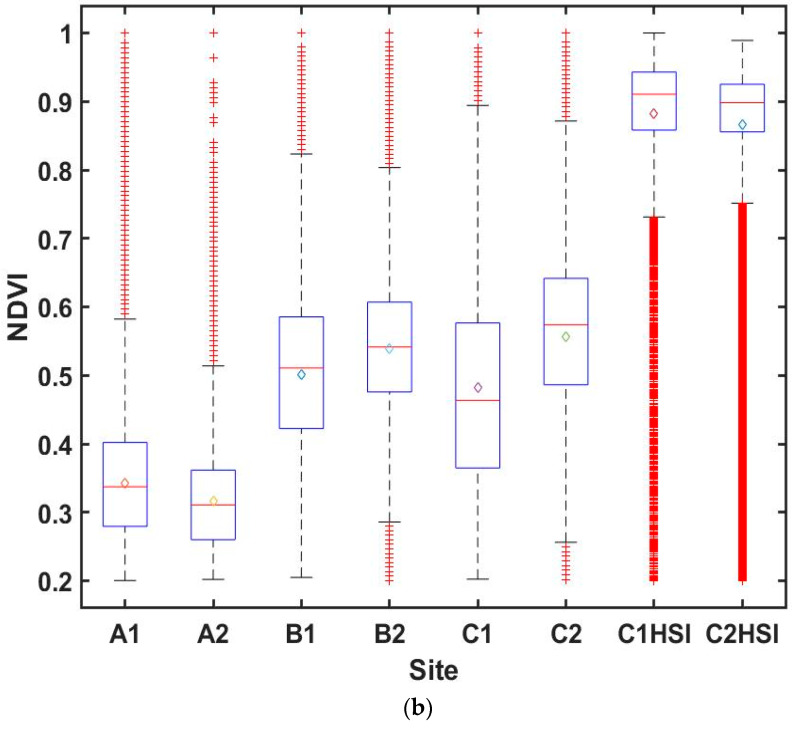
Box plots of the variables used for constructing the surface temperature against vegetation index (Ts-VI) spaces for all the sites. (**a**) Temperature; (**b**) normalized difference vegetation index (NDVI) (0.2–1.0). The bars on the box plots represent the distribution of the variables. The diamonds inside the boxes represent the mean values of the variables. The red marks beyond the short horizontal line are the outliers.

The box plots of the temperature and NDVI values (Figure 3) that were used to construct the Ts-VI spaces were generated to show their distribution. The results indicate that the mean values of the temperature, shown in Figure 3a, range between 14.69 °C (Site A2) and 23.02 °C (Site C2HSI); the mean values of the NDVI, shown in Figure 3b, range between 0.248 (Site A2) and 0.883 (Site C1HSI). When using the mean values to assess the temperature and NDVI differences, Site A (15.68 °C) is cooler than Site B (19.59 °C) and C (C1-2: 21.88 °C; C1HSI-C2HSI: 22.64 °C). A trend of Site A (0.331) having lower NDVI values than Site B (0.519) and C (C1-2: 0.519; C1HSI-C2HSI: 0.873) can also be observed. According to the mean values of each site, the NDVI value does not change linearly with the corresponding temperature value. 

According to the results of the Kolmogorov-Smirnov test (*p* = 0 for all the sites), the temperature and NDVI of each site are not normally distributed. Based on the nonnormal data distribution, we chose Spearman’s rank correlation coefficient Rs to calculate and assess the correlation between the two variables [56]. Results (Table 2) indicate a positive moderate correlation between the temperature and NDVI for the overall data, confirmed by the positive Rs value of 0.540 (*p* = 0; n = 5,718,215; 95% confidence level; two-tailed; [59]). However, the correlation coefficients vary between a weak correlation (i.e., | Rs | ≤ 0.35; [59]; Site A1, A2, C1, C2, C1HSI, and C2HSI) and a moderate correlation (i.e., 0.36 ≤ | Rs | ≤ 0.67; [59]; Site B1 and B2) in individual sites. Furthermore, Site B1-2, C1, and C1HSI are inversely correlated, whereas the other sites are positively correlated.

### 3.2. Ts-NDVI Spaces

**Figure 4:**
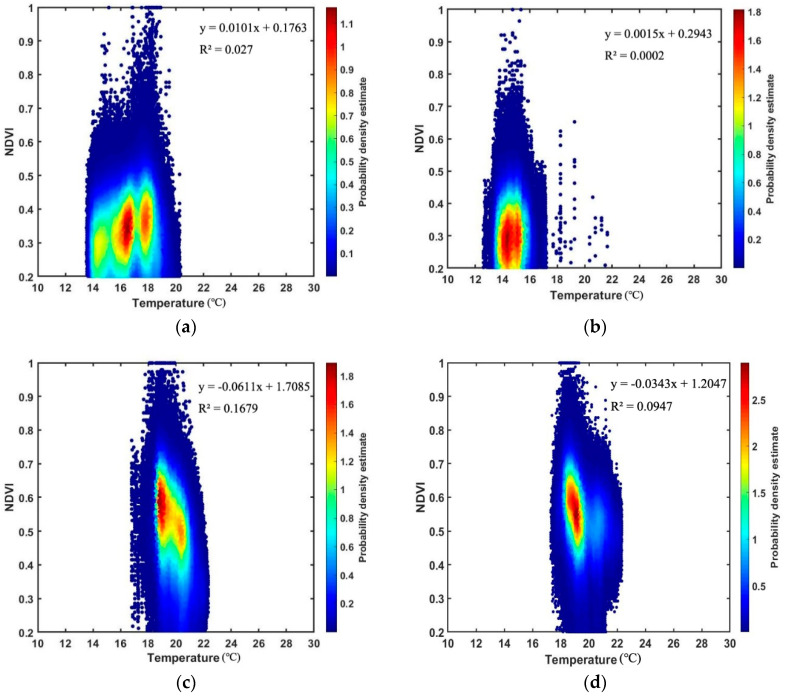

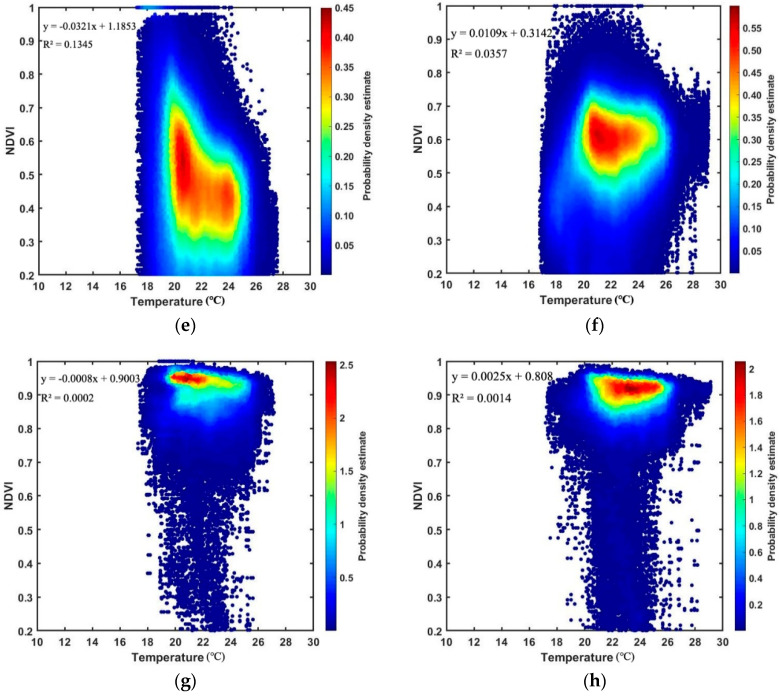
Ts-NDVI spaces of each site. (**a**) Site A1; (**b**) Site A2; (**c**) Site B1; (**d**) Site B2; (**e**) Site C1; (**f**) Site C2; (**g**) Site C1HSI; (**h**) Site C2HSI. The scatter points are marked in gradually-changed colors according to their densities calculated using a kernel smoothing function. For Site A1-2, B1-2, and C1-2, the NDVI values are calculated using the multispectral data. For Site C1HSI and C2HSI, the NDVI values are calculated using the hyperspectral data. All the NDVI values are limited to the range between 0.2 and 1.

The images of kernel density estimation (Figure 4) show the scatter plots of temperature values against the corresponding NDVI values in a two-dimensional space for all the sites. The gradually-changed colors in the images correspond to the density of each pixel or its neighbors [60]. Here, the ordinate of NDVI was limited to the range of 0.2–1 for all the sites, whereas the abscissa was limited to the range of 10–30 °C according to the temperature distribution of these sites. 

Our results show that the Ts-NDVI space differs in each plot. The scatter points of Site A1-2, B1-2, and C1-2 can form shapes such as a triangle or trapezoid in general, as described by Jiang and Islam [61] and Long and Singh [62]. In Site A1-2 and B1-2, the shape of the Ts-VI space resembles a truncated triangle, while in Site C1-2, the shape resembles a trapezoid. However, in Site C1HSI and C2HSI, the scatter points cannot form a triangular or trapezoid Ts-NDVI space.

Site A1-2, B1-2, and C1-2 present sharper regions at the upper parts of the Ts-NDVI spaces (Figure 4a–f). Two clear boundaries can be observed on the lower and higher temperature sides to limit the shape of the Ts-NDVI spaces of these sites. For Site C1HSI and C2HSI, the lower parts of the plots tend to be sharper with relatively sparse scatter points (Figure 4g–h). The dry and wet edges cannot be well observed for Site C1HSI and C2HSI due to the insufficient scatter points at the full range of NDVI.

### 3.3. Visualization of the Water-Stress Information

**Figure 5:**
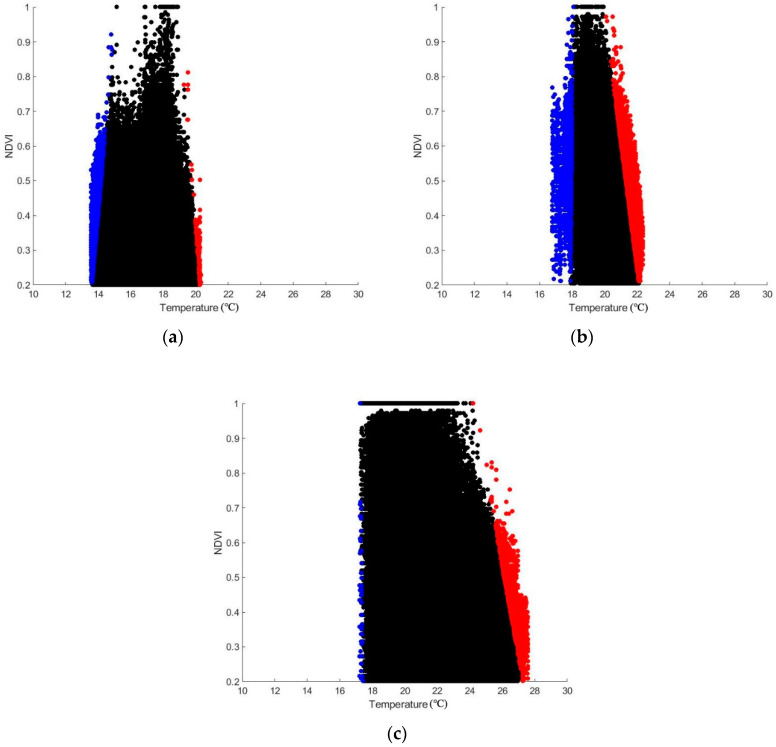
The dry and wet edges of the Ts-NDVI space. (**a**) Site A1; (**b**) Site B1; (**c**) Site C1. The points of the dry edge are in red color; the points of the wet edge are in blue color.

The algorithm described in Section 2.2.2 was applied to calculate the dry and wet edges in Site A1, B1, and C1 as examples. The result (Figure 5) displays the points of the dry and wet edges in red and blue, respectively. Due to the different shapes of the Ts-NDVI spaces, the number of the scatter points of the dry and wet edges varies in each site. The result shows that the dry and wet edges can form two physical limits of the Ts–NDVI triangle (trapezoid) space, as described by Margulis et al. [63]. According to Figure 5, the dry and wet edges can be well defined on the basis of the Ts-NDVI space using our algorithm. 

**Figure 6:**
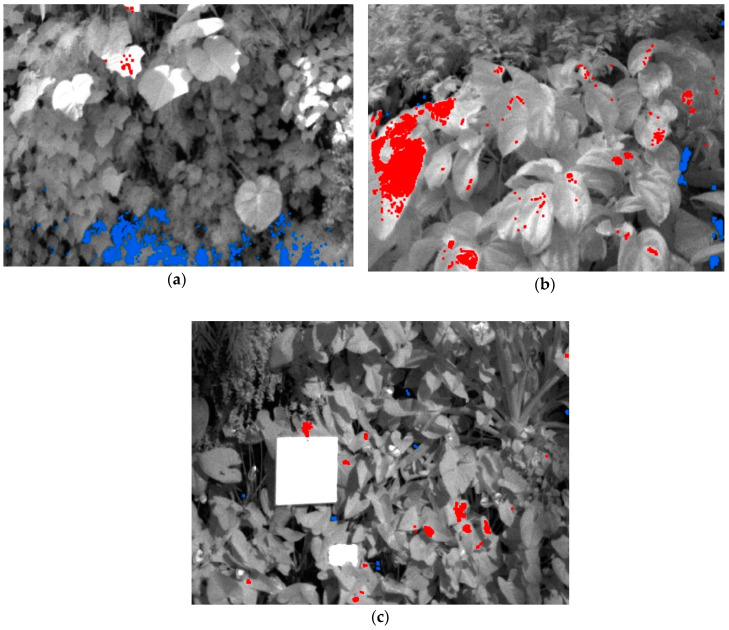
The visualization results of the area associated with dry and wet edges using the multispectral images. (**a**) Site A1; (**b**) Site B1; (**c**) Site C1. Pixels of the dry edge are in red; pixels of the wet edge are in blue. The multispectral images are displayed in the gray-color mode to highlight the colored pixels.

The results of the highlighted scatter points were then transferred to the multispectral images (Figure 6) to be directly displayed. Specifically, the pixels that corresponded to the scatter points of the dry and wet edges were marked in red and blue, respectively. To highlight the transferred results, the multispectral images were displayed in the gray-color mode. Based on the visual analysis, it can be concluded that the red pixels are exposed to the direct sunlight, whereas the blue pixels are generally located in the shade. The red and blue pixels on Figure 6 represent the areas with water-stress conditions and the areas with sufficient water conditions, respectively [24,31]. 

## 4. Discussion

Our study has documented the applicability of using the triangle method to monitor the water-stress of a living wall. To this end, we acquired thermal infrared, multispectral, and hyperspectral data in one of the largest living walls in the world: The Cloud Forest Conservatory of Gardens by the Bay in Singapore. Our results have provided a simple and effective method to obtain and directly display the water-stress information of the plants attached to a living wall using remote sensing. Furthermore, our findings suggest that the triangle method, which was developed for outdoor applications, works in an indoor environment.

Unlike previous studies, our data were acquired from an indoor environment fully enclosed by a transparent glass curtain wall, where temperature and humidity are controlled to emulate the climate of tropical mountain regions [64]. Each dataset covers a small area, meeting the requirement of homogenous atmospheric conditions that are necessary for the application of the triangle method [58]. The role of soil moisture for the triangle method was commonly highlighted in previous studies, but is not present in our study due to the research setting and the structure of the living wall described in Section 2.1. Our visualization results (Figure 6) indicate that the areas with water stress (in red) are mostly exposed to direct sunlight, whereas the areas with sufficient water conditions (in blue) are generally located in the shade or at the bottom of the wall. According to the study by Margulis et al. [65], who analyzed the water content of coffee plants under different levels of sunlight, higher-level exposure to sunlight results in lower water content in the plants. Direct sunlight can increase the temperature of leaves and the rate of evapotranspiration within a certain range, resulting in the acceleration of water loss and water stress in sunlit leaves. Therefore, our results are reasonable, validating the applicability of our method in monitoring the water stress of living walls. Moreover, the results suggest that the triangle method can be applied in an indoor environment. To further study the role of irrigation in such an environment, increased artificial irrigation could be carried out on the areas associated with the red pixels, i.e., the water-stress areas. 

In our study, the Ts-VI spaces were constructed using NDVI as the proxy for VI due to its simplicity [66]. The feature space differs in each plot (see Figure 4), which can be explained by the various vegetation types of the living wall related to the NDVI values [67] and the different ambient temperatures (see Table 1) related to the vegetation temperature values [68]. The Ts-VI spaces constructed using the NDVI data calculated from the multispectral data all result in shapes such as a triangle or trapezoid (see Figure 4), conforming to the previous studies of the triangle method (e.g., [69,70,71]). According to the study by Zhu et al. [25], the feature spaces do not form a regular triangle shape if there are no completely wet or dry surfaces in the images under the condition of uniform atmospheric conditions. In this context, it should be noted that there is no exposed soil on the living wall, and thus its direct effects on the Ts-VI spaces are minimal. 

The reliability of the Ts-VI triangle method lies in the determination of the dry and wet edges [25,72], especially the dry edge defining the maximum water stress [29]. In most previous studies, only the dry edge is determined by calculation [23], whereas the wet edge is set as a constant (e.g., the lowest observed clear pixel surface temperature [73], the lowest temperature at dense vegetation cover [72], the average remotely sensed inland water body temperature [34] or the average air temperature [74]). In general, the dry edges can be divided into two categories in the traditional triangle methods: One kind is the theoretical dry edge determined using the radiation budget and the energy balance equation [74,75]; the other kind is the observed dry edge determined by empirical methods based on statistical regression [76]. Here, we chose to determine the observed dry and wet edges according to the distribution of the scattered points. It was mainly because the theoretical method requires multiple inputs in surface and canopy resistance variables (e.g., wind speed, vapor pressure deficit (VPD), and air temperature; [39]), which deviates from the research setting of this study. Our algorithm of determining the dry and wet edges is inspired by the study conducted by Sun et al. [58] and Zhu et al. [28], in which the critical points of determining the two boundaries were elaborated. Similarly, four endpoints, determined by selecting the corresponding maximum or minimum temperature value for a given maximum or minimum NDVI value, were chosen to calculate the dry and wet edges in our study. 

Our algorithm of determining the dry and wet edges has three major advantages, as described in the following. First, our algorithm can provide a unique result of the observed dry and wet edges. In most previous studies, the results of the observed dry and wet edges may suffer from the uncertainty caused by subjectivity [24,26,29], as they are commonly determined using the statistical regression methods (e.g., [76]) and the wet edges using a constant (e.g., [74]). Here, we determined the dry and wet edges only by the temperature and NDVI values of the scatter points without any empirical relations, which can avoid the influence of subjectivity. Second, our algorithm is simple and effective with no requirement of complex calculations. The algorithms proposed in the previous studies commonly require multisteps of calculation (e.g., iterative process; [24,72,77]) to determine the dry and wet edge. In contrast, our algorithm only needs to sort the scatter points and find the four endpoints under the given temperature and NDVI values. As a high temporal resolution is important for monitoring a living wall, our algorithm is valuable due to its simplicity which enables high repeat visit frequencies. Thus, in-time measures could be carried out (e.g., artificial irrigation) to avoid the wilting of plants. According to the reasonable visualization results, the simplicity of our method does not compromise its accuracy in obtaining the dry and wet edges. Third, our algorithm can reduce the influence of noise points between the maximum and minimum VI values. In traditional methods, the dry edges were determined by calculating the selected pixels in each small VI interval using a linear regression [36,78]. Thus, noise points between the maximum and minimum VI values may affect the results. Unlike the aforesaid algorithms, our method is entirely based on the pixels at the maximum and minimum NDVI values, thus minimizing the influence of potential noise points in the determination of the dry and wet edges.

Similar to other models, our algorithm has its own limitations. The main weakness lies in the noise points or outliers with the maximum and minimum NDVI values, which may affect the determination of the observed dry and wet edges in the case of being selected as the endpoints. Moreover, due to the different shapes of the Ts-NDVI spaces, the pixel number of the dry and wet edges displayed in the visible images vary in different study areas. Thus, excessive or rare water-stress areas may be obtained from our algorithm, which can result in limitations of monitoring a living wall. Despite this, our method is still of significance because it has partially filled the knowledge gap that no workflow of monitoring the water stress of living walls has been established. Moreover, our study can be considered indirectly beneficial for the ecological environment due to the established environmental benefits of the living walls.

It should be noted that the dry and wet edges cannot be well observed from the Ts-NDVI spaces constructed by hyperspectral data, as they cannot form a triangular or trapezoid shape due to the deficiency of scatter points at lower NDVI values. This result conforms to the study by Sun et al. and Zhu et al. [25,58], who have elaborated that enough pixels at the full range of VIs are required in the application of the triangle method. Thus, when the range of different types of vegetation (e.g., healthy and not healthy) is limited or the spatial resolution of the remote sensing data is low, our method may be not applicable due to the insufficient pixels at the full range of the VI. The different shapes of the Ts-NDVI spaces constructed by the multispectral and hyperspectral data can be explained by the following. First, the bands used to calculate the NDVI between the two kinds of data are believed to be different. The bands used to calculate the NDVI from multispectral data are collected over a wider range of wavelengths, contrary to the hyperspectral bands that are recorded from a narrower range of wavelengths. Second, the pixel unmixing process was only conducted on the hyperspectral data to delete the pixels not associated with vegetation, resulting in the deficiency of scatter points with lower NDVI values.

Furthermore, our results suggest that the correlation between the NDVI and temperature vary in different sites of the living wall, according to the results of the Spearman’s rank correlation coefficient (see Table 2). As the vegetation species vary in different areas of the living wall, our finding conforms to the previous study by Chuai et al. [79], who also documented that different types of vegetation can result in different correlations between NDVI and temperature in the same area. 

This study was conducted in an indoor environment where visible light can be transmitted into the interior. Additional work based on the data acquired at an indoor environment where direct sunlight is blocked needs to be carried out to verify the applicability of the triangle method in this kind of an indoor environment. This is beyond the scope of our study and will not be discussed here. Further validation using spectral and biochemical leaf data, such as the leaf water content, stomatal conductance, and water evapotranspiration, could be beneficial to understand the degree of water-stress of this living wall system.

## 5. Conclusions

In general, the study presents a simple and effective approach to monitor the health status of an indoor living wall entirely based on remote sensing imagery and no empirical relations. Overall, our study suggests that the triangle method has the applicability to monitor the water stress of a living wall. The results can directly display water-stress information in a visually understandable manner that does not require skills or expertise in image analysis. A simple and effective algorithm was provided to determine the dry and wet edges, which can avoid the influence of uncertainty and the requirement of additional data sources. Unlike the previous studies, our data were acquired in an indoor environment that is fully enclosed by a transparent glass curtain wall, indicating that the triangle method can be transferred from outdoor applications to those inside a dome. It should be noted that outliers at the maximum and minimum NDVI values may be involved in the determination of the dry and wet edges, thus inducing inaccuracy in the results. Despite this, our method is valuable for the maintenance of living wall systems, and therefore significant to ecological environments. Our results and findings can extend the application scope of the triangle method in future studies. 

## Figures and Tables

**Table 1:** Parameters of the data acquisition at different sites.

Site	Air Temperature/°C	Relative Humidity/%	Distance/m	Time
A1	18.1	98.3	5.3	10:30:00 AM
A2	18.7	76.1	5.8	10:51:00 AM
B1	21.6	69.7	6.5	09:39:00 AM
B2	21.6	69.7	6.5	09:39:00 AM
C1	20.6	98.6	4.3	09:21:00 AM
C2	20.5	98.5	4.3	09:25:00 AM

**Table 2:** The Spearman’s rank correlation coefficient Rs between the temperature and NDVI at all the sites. N: Number of the points.

Site	Rs	P	N
A1	0.167	0.00	856,698
A2	0.027	0.00	695,458
B1	−0.404	0.00	918,844
B2	−0.367	0.00	788,834
C1	−0.342	0.00	796,454
C2	0.150	0.00	807,772
C1HSI	−0.107	0.00	340,384
C2HSI	0.002	0.09	513,771
All sites	0.540	0.00	5,718,215
